# Technical pen-based printhead for high-precision microdosing of personalized therapies

**DOI:** 10.1007/s13346-026-02065-1

**Published:** 2026-02-06

**Authors:** Paola Carou-Senra, Carlos Rial, Alex Richter, Abdul W. Basit, Carmen Alvarez-Lorenzo, Alvaro Goyanes

**Affiliations:** 1 Departamento de Farmacología, Farmacia y Tecnología Farmacéutica, I+D FarmaFacultad de Farmacia, Instituto de Materiales (iMATUS) and Health Research Institute of Santiago de Compostela (IDIS), Universidade de Santiago de Compostela, 15782 Santiago de Compostela, Spain; 2 FABRX Ltd., Henwood House, Henwood, Ashford, Kent TN24 8DH UK; 3 FABRX Artificial Intelligence, Calle Enrique Vidal Abascal 7, 15702 Santiago de Compostela, Spain; 4 Department of Pharmaceutics, UCL School of Pharmacy, University College London, 29-39 Brunswick Square, London, WC1N 1AX UK

**Keywords:** Technical printhead, High-precision microdosing, Additive Manufacturing, Personalized Medicine, Biologics, Low-dose drug delivery

## Abstract

Additive manufacturing offers unprecedented opportunities for personalized medicine, but most pharmaceutical printing platforms are optimized for milligram-range doses, limiting their suitability for microdosing. This work introduces a novel liquid deposition approach using a modified technical pen integrated into a pharmaceutical printer. The gravity-driven mechanism enabled precise microscale dispensing without external thermal, pneumatic, or electrical inputs, which have been associated with molecular stress in other printing technologies. Desmopressin, a potent synthetic hormone indicated for diabetes insipidus and requiring ultra-low doses, was selected as a model compound. Oral films (2 × 4 cm) containing therapeutically relevant doses (33–134 µg) were produced by depositing up to four layers of pharmaceutical ink. A custom-developed software interface allowed precise control of key process parameters, supporting reproducibility and automated workflows. The system achieved ~ 100% dose accuracy, with a strong correlation between drug content and layer number. Films exhibited rapid disintegration and immediate release. Stability testing showed no drug degradation over one month. Unlike more complex printing platforms, the technical printhead architecture offered straightforward manipulation and rapid setup. Given the constant ink flow rate and low, consistent, deposition volumes, only 1 mL of formulation is sufficient to produce up to 238 single-layer 2 × 4 cm films. These findings position the technical pen-based printhead as a promising, precise, and cost-effective addition to the additive manufacturing landscape, with strong potential for low-dose personalized pharmaceutical applications, including biologics. Moreover, its performance underscores the potential for further optimization and broader application.

**Graphical abstract:**

**Figure:**
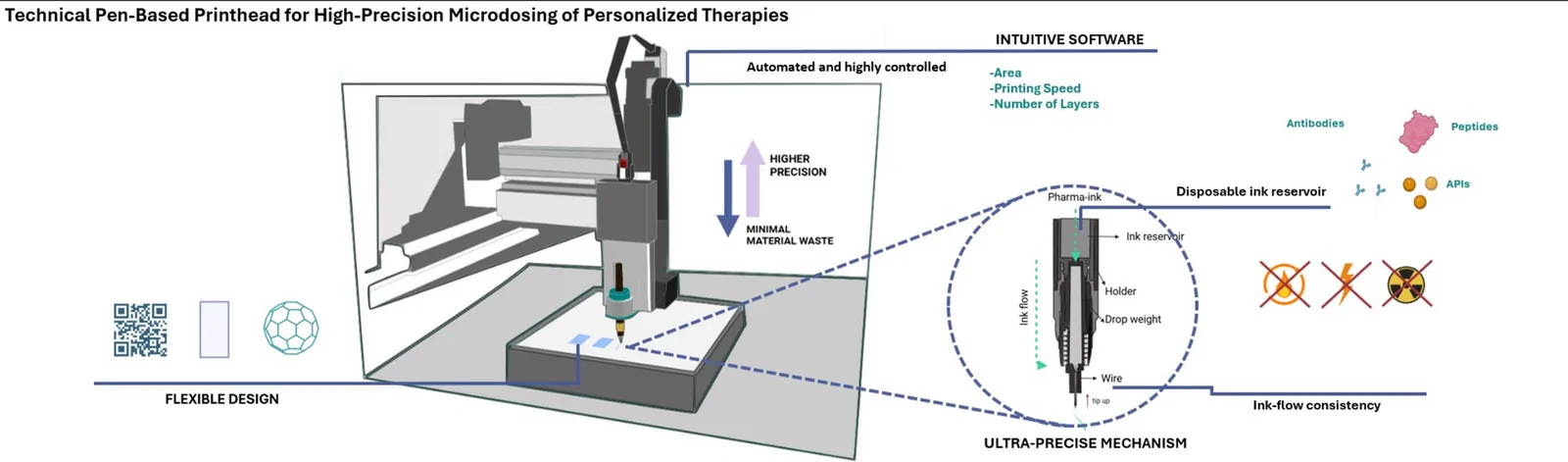

**Supplementary Information:**

The online version contains supplementary material available at https://doi.org/10.1007/s13346-026-02065-1.

## Introduction

Personalized medicine is transforming healthcare by individualizing treatments to patient’s needs [1]. Additive manufacturing is at the forefront of this revolution, allowing the on-demand production of pharmaceuticals, medical devices, and drug delivery systems with a high level of customization [2, 3]. Printing technologies make it now possible to combine multiple drugs in a single dosage form [4, 5], controlled release profiles [6, 7], and fine-tune treatments based on patient-specific needs[8].

As this field evolves, achieving precise control over material deposition in the microgram- or microliter-level is crucial, since accuracy is central for highly potent drugs and advanced drug delivery systems. An example can be found with some biological compounds, like desmopressin. Desmopressin is a well-known potent peptide hormone indicated for the treatment of diabetes insipidus [9]. Unlike the more common diabetes mellitus, which involves blood sugar regulation[10], diabetes insipidus is a rare endocrine disorder caused due to a deficiency in the production or action of vasopressin (antidiuretic hormone, ADH), a hormone essential for maintaining water homeostasis [11]. Treatment with desmopressin, the synthetic hormone that substitutes ADH effect, is prescribed in low doses (micrograms) and requires individualized therapy. Notably, studies have shown that women tend to be more responsive to its antidiuretic effects, often requiring lower doses than men [12]. Despite this, desmopressin is currently only available in fixed-dose formulations or liquid solutions[13], that could lead to dosing inaccuracy or stability issues. To avoid therapeutic inefficacy or safety risks in these sensitive applications, technologies that enable precise deposition at the micro-scale range are essential.

While 3D printing technologies offer versatile manufacturing capabilities, 2D printing methods such as inkjet printing (IJP) currently provide more precise control at liquid deposition of pharmaceuticals at very small doses [14]. IJP has emerged as a leading precision technology in this area, showing great promise due to its drop-by-drop (DoD) material deposition [14]. Numerous studies have been published involving the use of this technology for pharmaceutical applications, using desktop inkjet printers [15, 16] and, even, portable devices [17]. Despite the great potential this technology holds [18], the inner complexity of the system and ejection process slow down its implementation in clinical settings. This creates a growing need for developing a system with precise material deposition while simpler to implement at the point-of-care, ensuring easier compliance with GMP standards.

Technical pens are devices known for their precision and consistent line quality; they are commonly used for detailed work in architectural drawings and artistic illustrations. Their precision ink delivery is based on the gravity action to draw ink from a reservoir and transfer it to the tip [19]. Typically, a technical pen has a round chrome plated metal tube with a stainless-steel wire that dispenses uniform and consistent flow of ink, crucial for precise deposition. This deposition mechanism avoids the use of heating [20], radiation [21] or electrical pulses [22], as required by other additive technologies, thereby minimizing interaction with the ink. This is particularly advantageous when working with biological compounds, such as hormones, as it prevents exposure to stressful conditions and helps preserve their structure and functionality.

Technical pens also feature a refillable ink cartridge within their body. As ink is consumed, the cartridge can be refilled or replaced. These reservoirs are easily accessible, allowing for simpler monitoring and reducing the risk of cross-contamination due to easier cleaning and maintenance. The tip diameters can also be changed to fit different needs. The size of the nib determines the thickness of the lines, which also helps to control the amount of ink deposited [23]. This less complex and more accessible design not only would make the system cost-effective and easier to integrate in clinical settings but also could facilitate sterilization processes and compliance with GMP standards.

To fully harness the potential of technical pen devices, it is essential to automate the process in a way that seamlessly integrates precision and efficiency. The implementation of various additive technologies in the pharmaceutical field has led to the development of pharmaceutical printers with printheads designed for techniques such as semisolid extrusion (SSE) and direct powder deposition (DPE) [24, 25]. Engineering a printhead specifically tailored to technical pen mechanisms enables not only automation but also improved scalability, facilitating streamlined, high-throughput production.

In this study, a novel printhead based on a technical pen was designed, engineered, and integrated for the first time into a pharmaceutical printer (M3DIMAKER 2) to enable precise and flexible fabrication of oral films, loaded with a biological compound. A custom interface was also developed within the M3DIMAKER Studio software to allow real-time adjustment of key process parameters such as printing speed, number of layers, and printed area. Films (2 × 4 cm) containing up to four layers of desmopressin pharmaceutical formulation were manufactured. Drug content analysis was conducted to assess the process precision and dose accuracy. Furthermore, the drug release profiles of the printed films were thoroughly monitored, and disintegration tests were performed. Various techniques were employed to gain insights into the surface morphology and thermal properties of the oral films. Finally, stability tests were performed in the printed films to evaluate desmopressin integrity over one month storage.

## Materials and methods

### Materials

Desmopressin acetate (99.02%, analytical grade) purchased from Selleck Chem (TX, USA), 1,2 propylene glycol (PG, analytical grade, Ph. Eur.) purchased from Scharlau (Spain), methanol (≥ 99.8%, HPLC grade) purchased from Merck (Germany), ethanol (≥ 99.8%, analytical grade, Ph. Eur.) (VWR Chemicals, France), E-122 red colorant (Guinama, Spain), and bright blue colorant (Guinama, Spain) were reagent grade. Paracetamol (MW 151.16 g/mol, USP grade) was purchased from Sigma-Aldrich (Dorset, UK). Potato starch edible paper (0.3 mm thickness) was supplied by Decoración Dulce (Madrid, Spain). Na_2_HPO_4_ and NaCl (ACS grade) were obtained from Scharlab S.L. (Barcelona, Spain) and KH_2_PO_4_ (ACS grade) from ITW Reagents (Darmstadt, Germany). Acetonitrile (HPLC grade) was purchased from Merck (Darmstadt, Germany), while trifluoroacetic acid (TFA) (≥ 99.9% v/v, HPLC grade), was acquired from Fisher Scientific (Hampton, NH, USA).

### Pharma-ink formulation

Three drug-free base inks were developed and initially tested, each containing different percentages of ethanol:propylene glycol (EtOH:PG) 50:50 v/v%, 60:40 v/v% and 70:30 v/v%.

In addition, a pharmaceutical ink (pharma-ink) containing 8 mg/mL of paracetamol dissolved in EtOH:PG 70:30 v/v% was prepared and used for preliminary printing experiments aimed at evaluating printability, dose precision and the influence of printing speed on dose deposition.

A pharma-ink containing 8 mg/mL of desmopressin acetate dissolved in EtOH:PG 70:30 v/v% was subsequently used as the main formulation in this study to perform the primary experiments, following process optimization. Colourant (0.15% w/w) was also added to all formulations, included final pharma-ink, to make the printed patterns visible.

### Pharma-ink characterization

The drug-free base inks and the desmopressin pharma-ink were characterised based on its dynamic viscosity, and density.

#### Dynamic viscosity

Dynamic viscosity was measured in duplicate at 20 °C using a Rheologyst AR 1000-N Rheometer (TA Instruments, New Castle, DE, USA) equipped with a cone (ø 60 mm, angle 2.1°)-plate geometry. The inks (2 mL) were transferred to the Peltier plate and subjected to a shear rate ramp from 0.05 to 1000 s^−1^ in 5 min. The viscosity value of each ink was calculated as the mean value recorded at the highest shear rate value.

#### Density

The density was measured in duplicate with an ABT 220-4NM analytical balance (KERN & SOHN GmbH, Stuttgart, Germany). 250 µL of the ink were removed using a PIPETMAN L Fixed F1000L Gilson Pipette (Gilson Inc., Middleton, WI, USA) and accurately weighed at 20 °C.

### Printing process

**Fig. 1:**
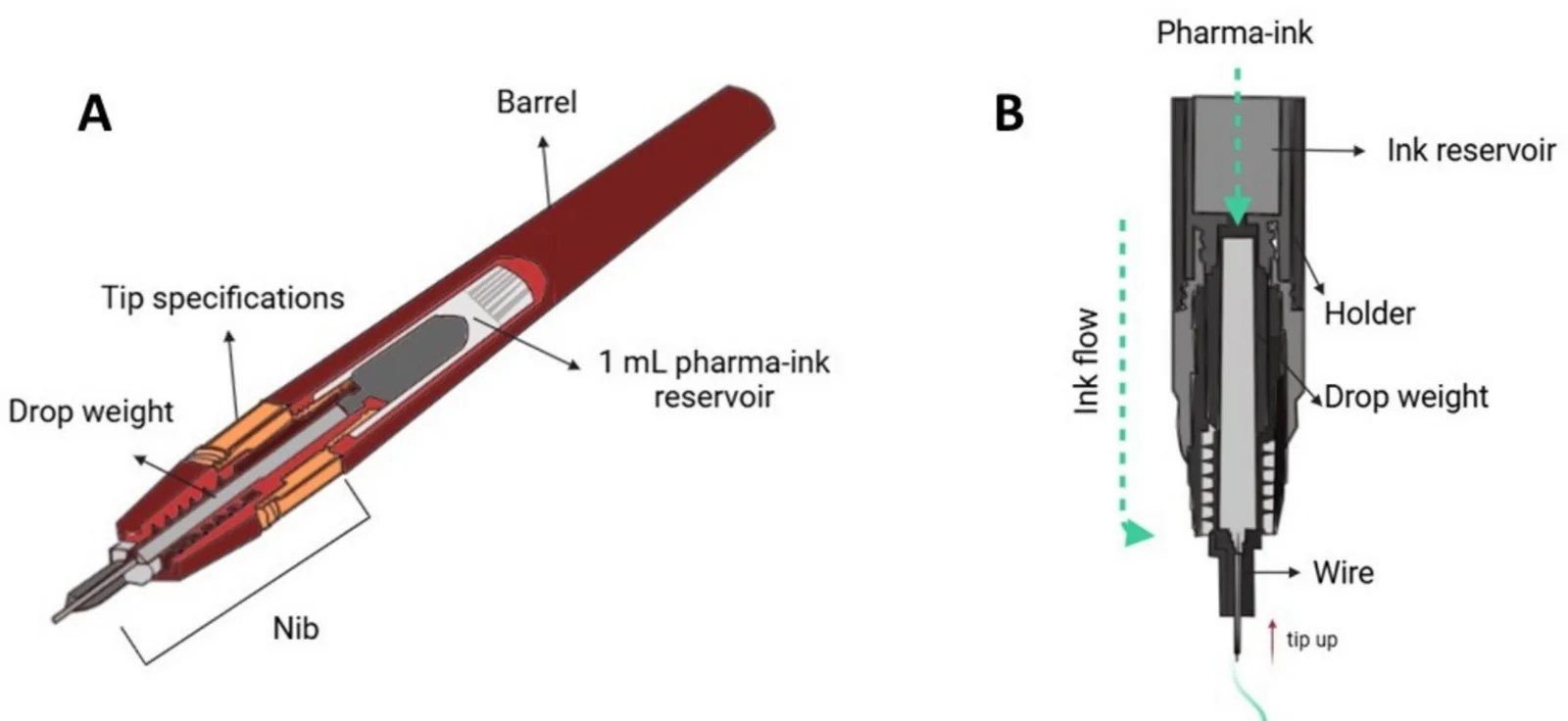
Rotring Isograph pen used for printing desmopressin onto edible films: **A**) image of the entire pen structure, with a cross-sectional view showing the internal components from the nib to the ink reservoir; and **B**) enlarged image of the internal structure of the nib

A Rotring Isograph technical pen (Rotring, Spain) was used to print pre-design figures containing the developed pharma-ink onto commercially available potato-starch edible paper (0.3 mm thickness). The Rotring pen consists of several components that ensure precise ink flow control (Fig. 1). Rotring Isograph pens are commonly protected with an outer casing that provides structure (Fig. 1A). Removing the outer casing, an ink reservoir can be found in the inner structure (Fig. 1B). For this study, the reservoir was emptied, cleaned, and sonicated using ethanol and distilled water. The reservoir was then refilled with pre-filtered (0.22 µm hydrophilic filters; Millipore Ltd., Dublin, Ireland) pharma-ink using a 1 mL disposable syringe. The reservoir supplies ink directly to the nib, creating a sealed system. The nib contains an internal wire that regulates the ink flow and prevents clogging, ensuring smooth and consistent lines. This thin, flexible metal wire inside the nib moves up slightly upon contact with the substrate, allowing ink to flow. Attached to the wire is a drop weight, i.e. a small cylindrical metal rod that moves slightly up and down in response to gravity and ink flow, further regulating the ink flow while preventing air bubbles and clogs inside the nib. The pen’s stainless-steel tip, available in various diameters, enables the creation of lines with consistent, pre-established widths.

**Fig. 2:**
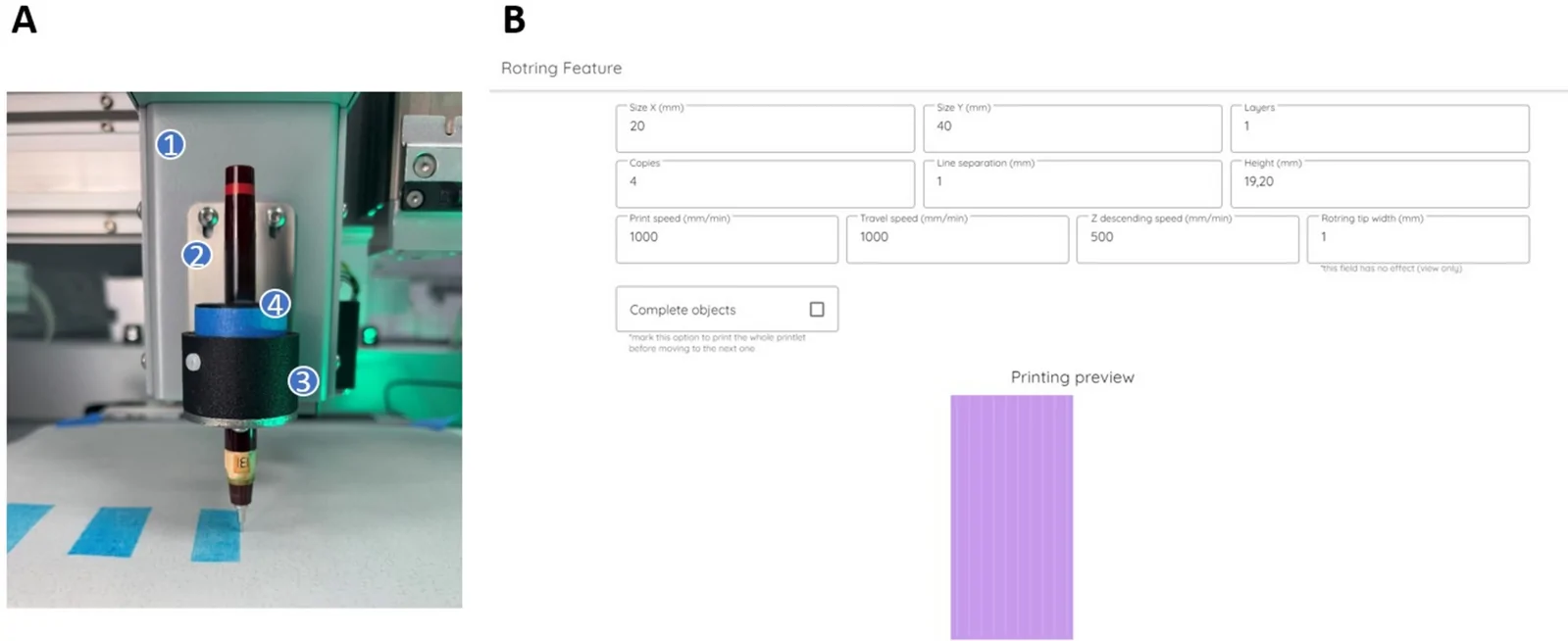
Images of **A**) the printhead assembly in use, featuring: (1) upper housing; (2) central mounting plate; (3) circular holder; and (4) modular adapter inserts. **B**) the developed custom interface in the M3DIMAKER Studio software

The printing process was carried out using the pharmaceutical 3D printer *M3DIMAKER 2* (FABRX Ltd., London, UK), equipped with a newly developed modular printhead specifically designed for this study. The printhead was engineered to enable the integration of a technical pen as the deposition tool. The assembly consists of a robust, upper housing which supports a central mounting plate. This plate includes a centrally positioned, machined circular holder. At the bottom of this circular holder, an internal ledge is integrated to act as a mechanical seat for modular adapter inserts. These circular adapters were precisely fabricated using Fused Deposition Modelling (FDM) 3D printing and were designed to fit tightly into the holder. Each adapter provides a form-fitting socket tailored to the outer diameter of the specific technical pen used. This modular approach ensures consistent axial positioning and firm radial retention of the pen, regardless of variations in pen housing dimensions. (Fig. 2A).

Additionally, a dedicated control interface was developed within the M3DIMAKER Studio software (FABRX Ltd.; London, UK) to operate the newly designed printhead (Fig. 2B). This custom module provided precise control over all critical printing parameters, including deposition area, layer count, printing speed, and tip width, ensuring consistent material output and fine spatial resolution. By allowing systematic adjustment and pre-setting of parameters, the software facilitated rapid optimization of new formulations and guaranteed reproducibility across multiple prints. The tight integration of hardware and software provided a robust and versatile platform, enabling precise control over dose and pattern geometry, which was essential when working with high-potency compounds.

In the printing process, rectangular patterns (2 × 4 cm) were used as the standard geometry for all experiments. As well, the line separation, i.e., the distance between each consecutive line drawn for the design, was set to 1 mm. The tip width use was 1 mm. The travel speed, which refers to the speed at which the printhead moves between printing multiple designs, was set to 1000 mm/min.

#### Print speed determination

The print speed, i.e., the speed at which the printer executes the design printing, was considered one of the most influential parameters in the printing process. To define the optimal print speed and to ensure the accuracy of the printing technology prior to the use of a high-potency biological compound, preliminary studies were conducted using a proof-of-concept pharma-ink containing 8 mg/mL paracetamol. Rectangular patterns (2 × 4 cm) were printed at two extreme printing speeds, 700 mm/min and 3000 mm/min, to assess both the accuracy and variability of the deposited material and to optimize printing parameters accordingly.

Following these preliminary experiments, the main experiments were subsequently performed using the pharma-ink containing 8 mg/mL desmopressin acetate. Rectangular patterns (2 × 4 cm) were printed at a printing speed of 1000 mm/min. Printing process was organized in batches of four objects and up to four layers. The software provided a “complete objects” option, which, when enabled, prints all layers of one object before proceeding to the next. If disabled, the first layer of all objects is printed, then the second layer, and so on. For this study, the latter option was chosen to facilitate drying between layers.

#### Ink flow rate determination

The ink flow rate was determined based on the experimentally printed dose, the known drug concentration in the pharma-ink, and the printing time for each condition (number of layers printed). For each printing condition, the experimentally determined drug dose was used to calculate the volume of ink dispensed, considering the drug concentration in the pharma-ink (8 mg/mL).

The ink flow rate was then calculated by dividing the dispensed ink volume by the corresponding printing time required to deposit the specified number of layers, according to Eq. 1.

1 $$Ink \;flow \;rate\; (mL/min)=\frac{(Experimental \;drug\; dose(mg))/Ink \;concentration\; (mg/mL)}{Printing \;time \;(min)}$$

This calculation was performed using a total of five replicates (*n* = 5) for each printing condition. An overall mean ink flow rate and corresponding standard deviation were calculated from these values.

### Characterization of the printed films

X-ray Powder Diffraction (XRPD) was performed in pure desmopressin, blank and desmopressin-printed films using a Bruker D8 Advance diffractometer (40 kV, 40 mA, theta/theta) (Bruker, Germany) equipped with a sealed Cu X-ray tube (CuKα1, λ = 1.5406 Å) and a LYNXEYE XE-T detector (Bruker, Massachusetts, USA). Diffractograms were recorded in the angular range of 3–40° with a step size of 0.02° and a counting time of 1 s per step. To assess whether any crystallinity could arise from components other than the drug–polymer interaction, XRPD analysis was also performed on control printed-films with up to four layers of drug-free EtOH:PG 70:30 v/v%. All tested samples were deposited onto an oriented crystal substrate (Si(511) plate) to minimize background scattering typically associated with glass-based supports. For the mathematical analysis of the obtained diffractograms and to overlay comparison patterns, the software HighScore Plus V.3.0 was used.

Scanning electron microscopy (SEM) was used to visualize the surface of drug-printed and blank films analysed using a Zeiss EVO LS15 scanning electron microscope equipped with a backscattered electron detector (Oberkochen, Germany), operating at 20 kV.

Thermal gravimetric analysis (TGA) runs were recorded under nitrogen atmosphere (60 mL/min) in a TA Discovery 55 (TA Instruments, New Castle, DE, USA). The samples (2.6–2.8 mg) were heated from 25° to 350 °C at a rate of 10 °C/min and the changes in mass were recorded and analyzed using a Trios v.4.2.1.36612 software.

### Drug content analysis

Desmopressin-printed films were placed in a volumetric flask (20 mL) with Milli-Q water under magnetic stirring at 200 rpm overnight. Then, 2 mL aliquots were taken, prefiltered through 0.22 µm hydrophilic filters Millipore Ltd., Dublin, Ireland) and centrifuged at 14,000 rpm for 45 min at 25 °C. Once the samples were centrifuged, the supernatant was collected and filtered through 0.22 µm hydrophilic filters (Millipore Ltd., Dublin, Ireland). Desmopressin quantification was determined using High-Performance Liquid Chromatography (HPLC) (JASCO LC-4000 Series, Jasco, Spain). The stationary phase was a C18 5 µm 4.6 × 250 mm column (Waters, Milford, MA, USA). The method for desmopressin analysis has been adapted from a previously reported study[26], the mobile phase consisted of 75:25 (v/v) 0.1% TFA Milli-Q water-acetonitrile. The flow rate was set on 0.8 mL/min with a column temperature of 30 °C and an injection volume of 70 µL. Quantification was measured at a wavelength of 220 nm and the retention time was 4.5 min. Results were reported in mean dose ± SD (*n* = 5). Dose accuracy was calculated as Eq. 2:

2 $$Dose \;Accuracy\; \left(\%\right)=\frac{Experimental \;Dose \;(\mu g)}{Estimated \;Dose\; (\mu g) } \times 100$$

The estimated dose was defined as the theoretical amount expected for a given area and number of layers. The measured dose for a 2 × 4 cm film with one layer was used as the reference. Based on this value, estimated doses for additional layers were calculated proportionally, ensuring consistent µg/cm^2^ deposition across all conditions; for example, the dose for two layers was expected to be twice that of one layer.

Printed films (2 × 4 cm) containing 1 layer of the proof-of-concept pharma-ink with 8 mg/mL paracetamol were processed in the same way as described above for desmopressin prior to HPLC analysis. Paracetamol quantification was performed using a validated HPLC assay from a previously reported study [27]. 20 µL of sample were injected into a Waters Spherisorb 5 µm C8 column (4.6 mm × 250 mm; Waters, Milford, MA, USA), with separation achieved using a mobile phase composed of water (85% v/v) and methanol (15% v/v) at a flow rate of 1 mL/min, column temperature of 40 °C, and detection at 247 nm.

### In vitro drug dissolution

Desmopressin-loaded printed films (2 × 4 cm) produced using 1-layer and 4-layers of pharma-ink were placed into plastic clear vials with pre-warmed (37 °C) 20 mL of simulated saliva (pH = 6.75 ± 0.05). The simulated saliva (pH = 6.75 ± 0.05) was prepared by dissolving 2.38 g Na_2_HPO_4_, 0.19 g KH_2_PO_4_ and 8.00 g NaCl in 1000 mL of distilled water [28]. The vials were placed in a shaker maintained at a temperature of 37 ± 0.5 °C and shaking speed of 100 rpm. At predetermined time intervals, 500 µL aliquots were withdrawn and replaced with fresh medium to ensure sink conditions. Aliquots were centrifuged at 14,000 rpm, 25 °C for 45 min. The supernatant was extracted and filtered through 0.22 µm filters (Millipore Ltd., Dublin, Ireland). The study was extended up to 2.5 h to ensure complete dissolution. The tests were carried out in triplicate for 1 and 4-layers printed-films. Desmopressin released amounts were quantified using the HPLC method described in Sect. "Drug content analysis".

### Disintegration testing

Disintegration time was evaluated to assess the mechanical breakdown of the developed oral films using the slide frame method. Printed films (2 × 4 cm) were placed flat over the opening of a 25 mL beaker, fully covering it. 200 µL of simulated saliva (previously heated at 37 °C) was added centrically on the film surface using a micropipette. The time required for the simulated saliva to wet the film matrix until the first drop falls into the beaker was measured. Measurements were performed in duplicate (*n* = 2).

A One-Way ANOVA was used to analyse whether there were significant differences in the disintegration times for the different printed layers, ranging from 0 to 4 layers (p < 0.05). Statistical analyses were conducted using GraphPad (GraphPad Prism 2019, GraphPad Software, Inc, CA, USA).

### Stability test

Stability studies were conducted over a one-month period on the films printed with 1, 2, 3 and 4-layers. For each one of the printed layers, five printed films from five different batches were stored in sealed containers and protected from light and humidity. After 1-month period, printed films were analysed to evaluate their dose content and the percentage of the theoretical dose, as described in Sect. "Drug content analysis" (*n* = 5).

## Results and discussion

### Drug printing and film characterization

**Fig. 3:**
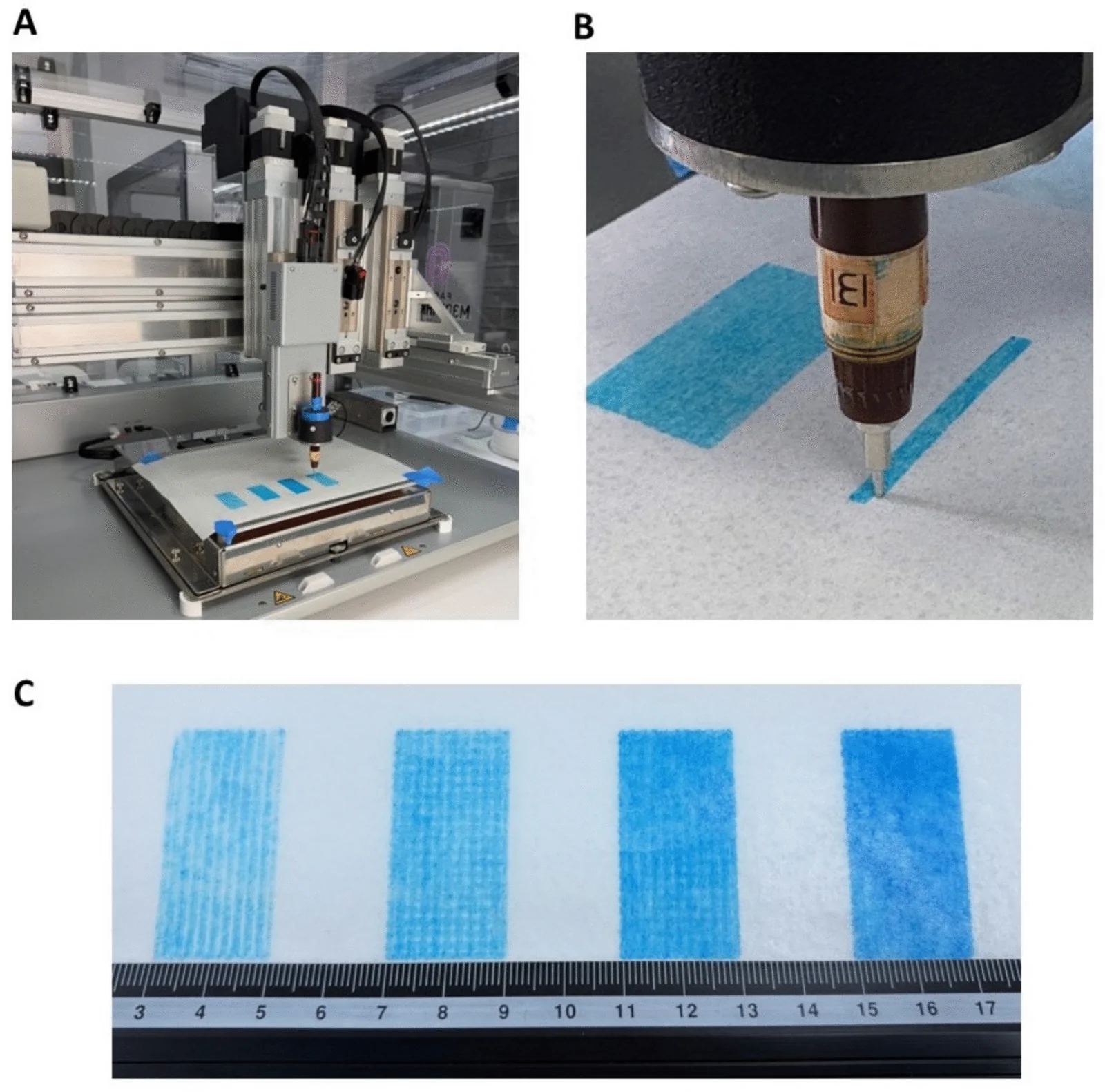
Printed films with up to 4-layers using pen printhead using EtOH:PG (70:30) and desmopressin pharma-ink with a blue colorant: **A**) image of the printing process of a batch of 3-layer films; **B**) image of the printing process focused on the tip of the pen; and **C**) image of the printed films from left to right: 1-layer film, 2-layer film, 3-layer film and 4 layer-film

For the first time, a technical pen was adapted and implemented as a novel printhead for pharmaceutical 3D printers, enabling the development of oral films containing a biological compound. The dedicated interface module integrated into the M3DIMAKER Studio software enabled precise control over design parameters, allowing the definition and execution of a 2 × 4 cm rectangular pattern. Using this interface, up to four layers of desmopressin-loaded pharma-ink were accurately deposited onto the commercial substrate (Fig. 3).

To ensure the correct deposition of the pharma-ink loaded with desmopressin, firstly different drug-free base formulations prepared with EtOH:PG mixtures at different percentages were evaluated. The EtOH:PG 50:50 v/v % and 60:40 v/v % mixtures had slightly higher viscosities and densities than EtOH:PG 70:30 v/v%, resulting in a more irregular and interrupted flow (Table 1). In contrast, EtOH:PG 70:30 v/v% demonstrated a smoother and uninterrupted deposition, consequently being selected as the base formulation to incorporate the drug in a concentration of 8 mg/mL and used for further studies.

**Table 1:** Developed drug-free base inks and pharma-ink, rheological properties and experimental printability

Formulation	Density (g/mL)	Viscosity (mPa·s)	Printability (Yes or Irregular flow)
EtOH:PG (70:30) and desmopressin	0.83 ± 0.01	2.21	Yes
EtOH:PG (70:30)	0.81 ± 0.02	2.77	Yes
EtOH:PG (60:40)	0.85 ± 0.03	2.93	Irregular flow
EtOH:PG (50:50)	0.90 ± 0.02	4.83	Irregular flow

Through the developed interface of the software, key printing parameters were fine-tuned during preliminary optimization, with a primary focus on printing speed. Preliminary studies using 1-layer films of the proof-of-concept pharma-ink containing 8 mg/mL paracetamol demonstrated that printing speed had a significant effect on the amount of drug deposited. At a high speed of 3000 mm/min, the drug content of 1-layer was 16.20 ± 1.04 µg per 2 × 4 cm film, whereas at a lower speed of 700 mm/min, the deposited amount increased to 35.38 ± 0.29 µg, accompanied by reduced variability between printed figures (*n* = 3). These results indicated that higher speeds led to lower deposition. Consequently, very high printing speeds were initially discarded, and a compromise speed of 1000 mm/min was chosen, balancing printing time with sufficient drug deposition, which was especially important given the high potency of the compound used in the main study. Batches of four figures were printed in each one of the printing processes. Printing a 1-layer batch took approximately 5.5 min, while printing a 4-layer batch took about 21.5 min (Table 2). Shorter printing times can still be achieved by increasing the printing speed.

**Table 2:** Number of deposited ink layers, desmopressin acetate dose, dose accuracy (%), and disintegration time (s) of 2 × 4 cm printed films using EtOH:PG (70:30) and desmopressin (8 mg/mL). Mean values ± standard deviations (*n* = 5)

Number of layers	Printing time (s)*	Dose (µg)	Dose accuracy (%)	Disintegration time (s)
0-Layer	-	-	-	4.68 ± 0.20
1-Layer	86	33.56 ± 1.10	-	4.82 ± 2.55
2-Layer	165	66.80 ± 1.05	99.53	5.41 ± 3.38
3-Layer	240	100.90 ± 0.72	100.21	6.68 ± 2.09
4-Layer	325	134.34 ± 1.24	100.08	9.88 ± 1.95

*In each printing process, 4 figures were printed per layer. The printing time (s) for each figure based on its layers was estimated by dividing the total duration by 4

The printed doses of desmopressin ranged from 33 µg to 134 µg, offering a flexible approach to dosing for various therapeutic requirements (Table 2). For diabetes insipidus, maintenance treatment ranges from 0.1 mg administered in three separate doses (~ 33 µg per dose) to 0.8–1.2 mg administered in two separate doses [29]. By simply varying the number of printed layers, therapeutically relevant doses were achieved. Furthermore, the dose accuracy between 99.53–100.21% demonstrated high level of precision in the dose printed. In this way, a linear relationship can be established between the dose and the area and number of layers printed. This allows the desired doses to be obtained by simply calculating these two parameters beforehand and modifying the values in the software. In fact, currently marketed oral tablets offer fixed doses of 0.1 and 0.2 mg [30, 31]. Using the implemented printhead, low and precise dosages could be achieved. Higher doses can be obtained simply by increasing the printed area, number of layers or pharma-ink concentration.

By performing the corresponding calculations for the different printing conditions, particularly considering the number of printed layers, a mean ink flow rate of 3.055 ± 0.094 µL/min was obtained. These results indicate that the ink flow rate remains constant and well controlled, even when multiple layers are printed. As a consequence of this stable flow behaviour, the average volume deposited per layer for a 2 × 4 cm printed area was 4.193 ± 0.012 µL. As demonstrated in previous studies using technical pens, ink deposition is influenced by multiple interrelated factors, including the nature of the substrate and its absorption capacity, the line width, the deposition speed—where higher speeds have been shown to reduce ink uptake—as well as ink properties [32]. Based on the stable ink consumption achieved in this study, it can be estimated that approximately 238 individual 2 × 4 cm single-layer prints can be produced using only 1 mL of ink, while up to 59 prints of the same area can be fabricated when four layers are deposited. Overall, these results demonstrate a high printing efficiency, characterized by low ink and drug consumption, as well as excellent control over ink delivery, which is particularly advantageous for pharmaceutical applications where material availability and dosage precision are critical.

**Fig. 4:**
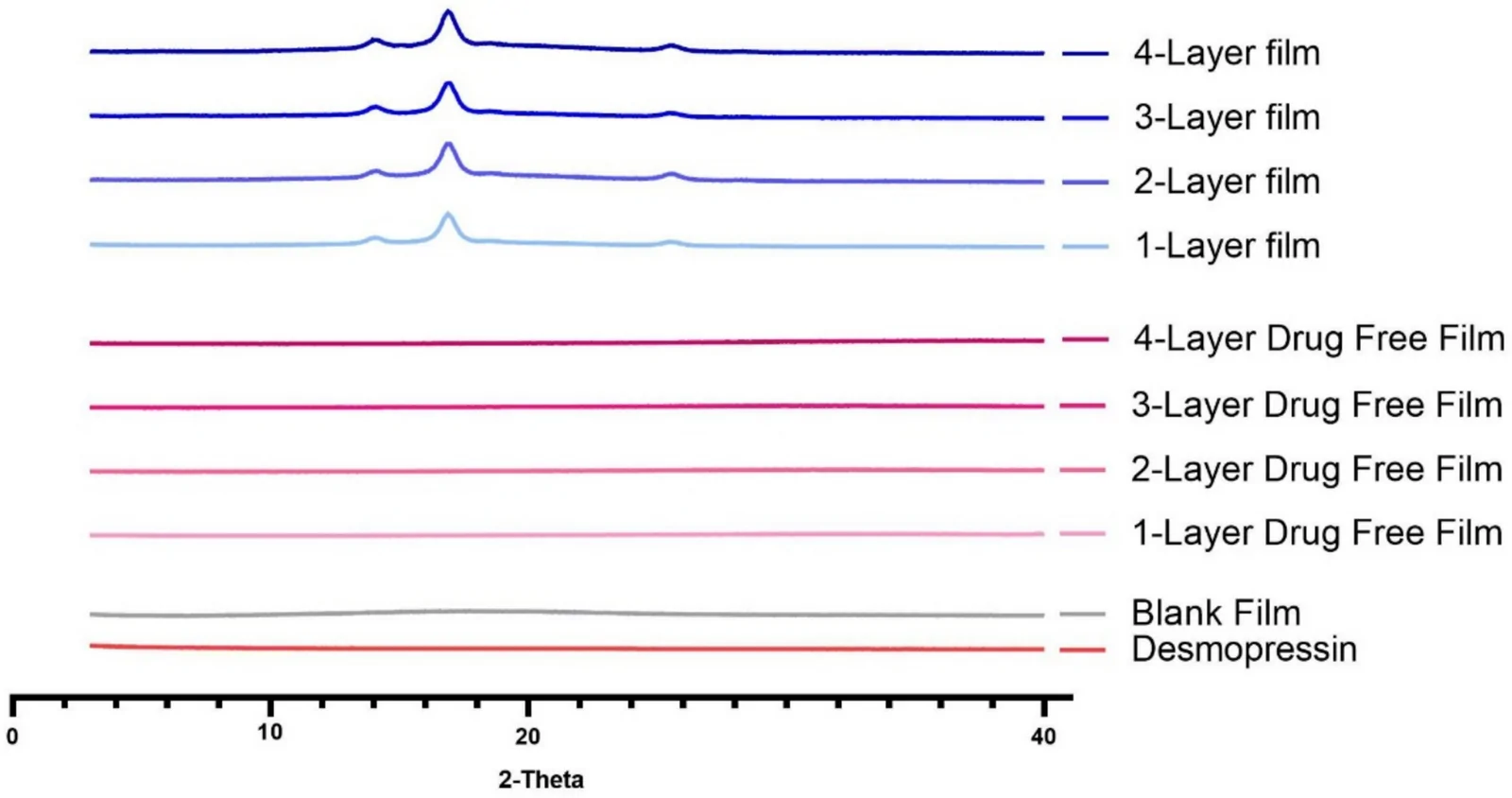
X-ray powder diffractograms of pure desmopressin, blank film and printed films with the pharma-ink (EtOH:PG 70:30 v/v% and desmopressin) and drug-free ink (EtOH:PG 70:30 v/v%) up-to 4 -layers

X-ray diffraction (XRD) was performed to study the crystallinity in the drug-loaded films and in the pure components. Pure desmopressin powder was in an amorphous state, as was the non-printed (Fig. 4). However, with the deposition of printed layers, crystallinity peaks began to emerge. Notably, three peaks were observed at 14.06°, 16.88° and 25.53°. These peaks were the result of the interaction between the pharma-ink and the printing substrate, which led to the rearrangement of molecules into crystals. The peaks observed in the different printed samples had similar widths, indicating their link to the same species, which reflected a drug-substrate interaction. These peaks increased with the number of layers deposited into the substrate, resulting in the highest peaks observed in the 4-layer films. Recent studies on desmopressin with other polymers, such as sodium polystyrene sulfonate (NaPSS), have also demonstrated structural rearrangements leading to crystalline organization [33]. Furthermore, no peaks were observed in the control films (printed with drug-free ink), supporting the hypothesis that crystalline complexes are formed as a result of drug–substrate interactions. This reinforces the idea that desmopressin could undergo molecular reorganization upon interaction with specific polymers, which may explain the appearance of some crystallinity peaks when applied to a starch-based substrate after solvent evaporation. Moreover, at 20.17°, a small band was observed which could be related to the substrate, that could also be observed in the blank film (Fig. 4).

**Fig. 5:**
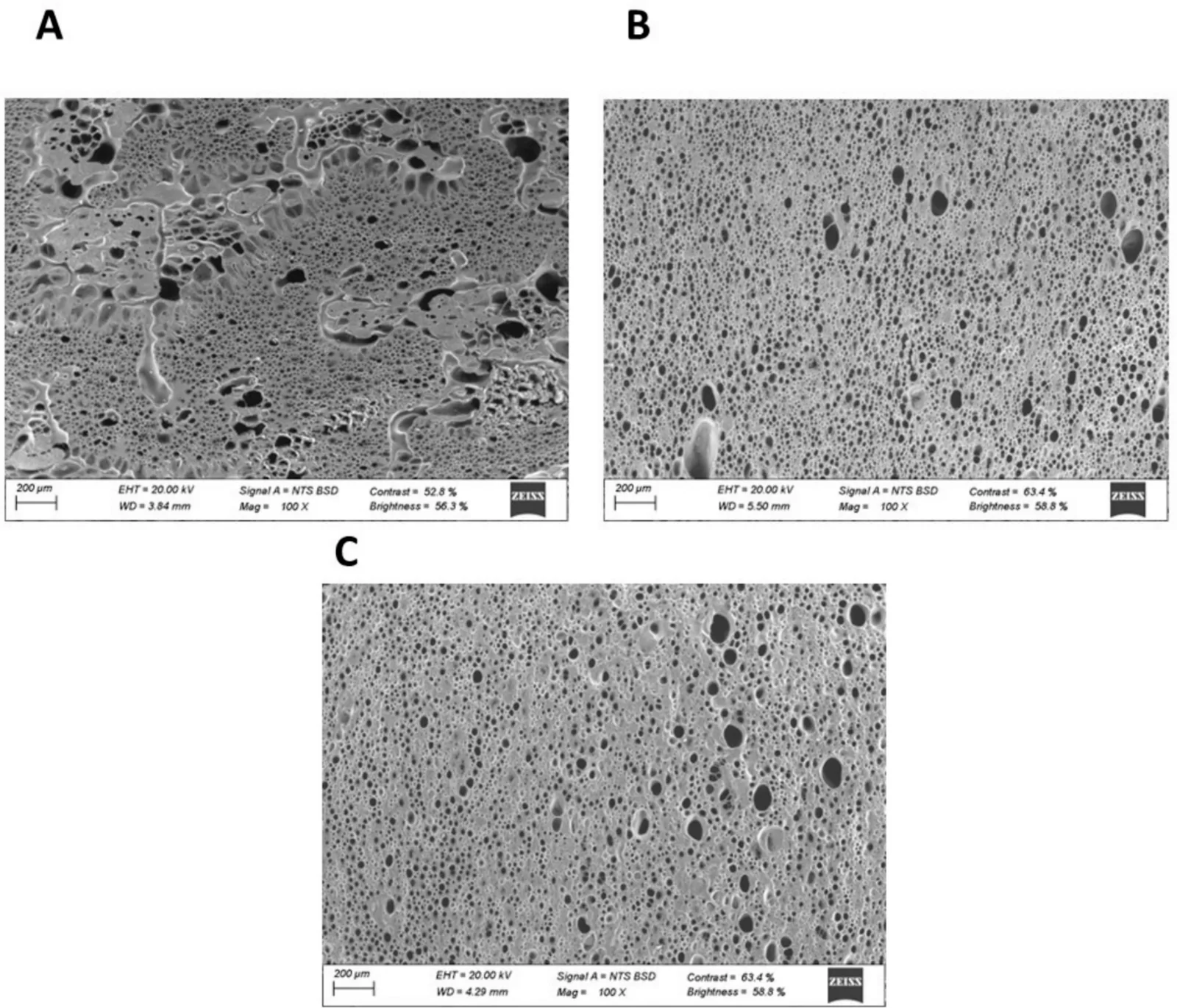
SEM images of the surface of: **a**) Blank film, **b**) 1-layer printed film, and **c**) 4-layer printed film with desmopressin pharma-ink EtOH:PG (70:30) and desmopressin

SEM was used to analyse the surface morphology of the films. The drug-free film exhibited a much rougher surface with more pronounced and irregular pores, approx. 115 μm of diameter, as well as larger protuberances (Fig. 5A). In contrast, drug-loaded films displayed significantly smaller and more uniform pores, lacking the prominent protuberances observed in the blank film (Fig. 5B and 5 C). Although some pores of approximately 109 μm could still be observed, the overall structure was significantly more uniform, consisting mainly of smaller pores with an average diameter of approx. 28 μm.

TGA scans of drug-free film and 4-layers film were very similar. The weight loss at 100–200 °C was related to the moisture loss and was slightly higher in the drug-free film compared to the 4-layer printed film (Figure S1). However, this could be attributed to a higher moisture content in the drug-free film. The more pronounced pores in the surface may facilitate greater moisture retention. The second stage, 250 < T < 350 °C, was related to the main degradation of the potato starch. The findings align previous reports on potato starch edible paper [34].

### Drug release, film disintegration and formulation stability

**Fig. 6:**
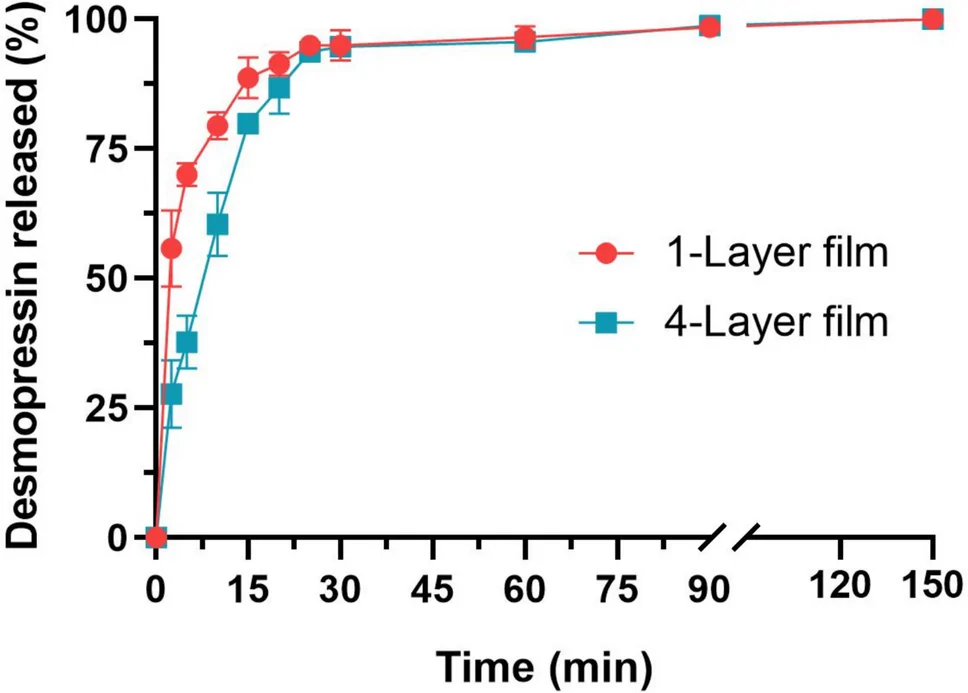
Dissolution profiles of desmopressin acetate from 1-Layer and 4-Layer printed films with pharma-ink EtOH:PG (70:30) and desmopressin in SSF (pH = 6.8) at 37 °C

Dissolutions tests were conducted to evaluate the release pattern of desmopressin from the drug-printed films. The films exhibited rapid release, with more than 50% of desmopressin dissolving within the first 10 min (Fig. 6). Over 90% of the drug was released before reaching 25 min, while the remaining 10% was released during the following hour. Dissolution was slightly faster for the 1-layer printed film.

Disintegration times for both blank and printed films were quite short, ranging from 4 to 10 s, with a slight increase observed as the number of layers increased (Table 2). The printing of different layers and their subsequent drying could possibly lead to an increase in disintegration time, as these layers create a hydrophobic barrier that the saliva droplet must penetrate before the film breaks.

After 1-month of storage, printed films exhibited dose accuracies around 100%, regardless the number of printed layers (Table 3). Drug contents were similar to those measured 1-day after printing. This demonstrates the stability of these films over time, confirming their reliability for long-term use. These findings suggest that even being an on-demand technology, printed-films produced could be suitable for practical applications even after extended storage periods.

**Table 3:** Number of layers of the deposited pharma-ink, the drug dose and dose accuracy after 1-month (%) storage of printed-films. Results reported as mean values ± standard deviations (*n* = 5)

Number of layers	Dose after one month (µg)	Dose accuracy after one month (%)
1-Layer film	34.68 ± 0.92	103.36 ± 2.74
2-Layer film	66.15 ± 1.06	98.56 ± 1.59
3-Layer film	100.44 ± 2.82	99.77 ± 2.80
4-Layer film	133.81 ± 2.45	99.68 ± 1.83

For the first time, a technical pen was engineered and implemented as a novel printhead within a pharmaceutical printing platform to enable the fabrication of low-dose desmopressin-loaded films. Achieving accurate low-dose formulations remains a major challenge in pharmaceutical manufacturing due to the high level of control required. Common 3D printing technologies, such as semisolid extrusion (SSE) and Direct Powder Deposition (DPE), typically deposit larger amounts of material, resulting in printlets weighing several milligrams [24, 35]. In addition, these technologies rely on temperature-assisted processes for material deposition, which may limit their applicability for thermolabile compounds. Direct Ink Writing (DIW) represents another 3D extrusion-based printing technique, distinguished from SSE by its higher spatial resolution and different deposition mechanism [36]. DIW relies on pressure-driven, layer-by-layer deposition of viscoelastic inks through fine nozzles, enabling printing with meso- to microscale precision [37, 38]. DIW has emerged as a highly valuable technology for the fabrication of complex 3D architectures and has therefore been widely explored in fields such as soft robotics [39], electronics [40], and biomedical engineering, particularly for implants, medical devices, wearables, and tissue-engineered constructs [41–43]. Despite its precision, DIW often requires post-printing consolidation steps to ensure structural integrity, increasing production time and energy demands [36]. Moreover, printing fidelity in DIW is strongly governed by ink rheology and nozzle diameter. While smaller nozzle diameters can improve resolution, they require increased extrusion pressures and longer printing times [36]. The pressure-driven flow and shear stresses within the nozzle have also been shown to affect material integrity, especially for bioprinting applications, highlighting the sensitivity of this process to mechanical stresses [44].

As discussed, currently 3D printing technologies, despite their high precision, are primarily oriented toward building 3D structures layer-by-layer, often requiring extended printing times, external energy, and sometimes post-processing. In contrast, 2D printing approaches can address a complementary space in pharmaceutical development, enabling the deposition of pharmaceutical formulations in low volumes for fine control over dose and narrower ranges.

In this context, IJP is among the most recognized and promising techniques for micro-deposition in 2D pharmaceutical applications. Its DoD modality, piezoelectric or thermal printheads, allows controlled volumes of pharma-ink to be deposited onto a variety of substrates, and offers remarkable versatility for the fabrication of different dosage forms [14]. This capability has made IJP widely explored in personalized pharmaceuticals for drug deposition onto films, contact lenses, microneedles or even medical devices [16, 17, 45]. However, the implementation of IJP in clinical settings faces several challenges, primarily related to the complexity of fluid handling. The ink passes through multiple stages and internal components- including reservoirs, connecting tubes and pumps, printhead channels, and the nozzle itself- before being ejected [46]. This complex fluid path can result in drug adsorption to surfaces, jet break-up, and variability in droplet volume. Once the ink reaches the internal channels feeding the nozzles, additional challenges arise, such as in-flight droplet deviation due to aerodynamic effects and differences in morphology, trajectory, velocity, and volume between the first and subsequent drops [47, 48]. Moreover, nozzle clogging, cleaning difficulties, and the management of dead volumes present further practical barriers, as disassembling and validating these printhead is laborious and costly. Therefore, while inkjet printing (IJP) continues to hold significant promise for pharmaceutical applications, alternative printhead designs with reduced fluidic complexity may offer complementary advantages, particularly in terms of ease of implementation, while helping to mitigate some of the practical challenges associated with more complex systems.

The developed technical pen-based printhead provides a complementary approach to established 2D micro-deposition techniques such as IJP. Its streamlined internal design enables a direct connection between the reservoir and a steel wire, eliminating complex fluid pathways, pumps, and internal channels. This simplified flow path minimizes dead volumes and reduces the risk of jet break, allowing the liquid to be dispensed uniformly solely upon contact with the substrate. The flow from the reservoir to the tip is gravity-driven, requiring no external pressure, thermal, or electrical impulses. This external energy inputs have been associated with molecular stress, so is expected that this novel gravity-driven mechanism provides a gentler environment for sensitive molecules. As the printhead design advances, future studies will be directed toward a more detailed evaluation of shear conditions within the system, enabling an even more precise positioning of this technology.

Importantly, the ink flow rate was shown to be consistent across different numbers of layers and at different stages of printing, which translated into a precise and controlled deposition. Two pharmaceutical compounds of markedly different nature were used in this work. In preliminary studies, paracetamol exhibited low variability in deposited dose even when different printing speeds were applied. In parallel, the model drug, desmopressin, was successfully deposited, within multiple layers of pharma-ink containing microgram-level quantities (33–134 µg) of clinical relevance, onto biocompatible starch-based substrates, achieving a dose accuracy near 100% with high reproducibility. Even after 1-month storage, desmopressin-films preserved its integrity and drug content, demonstrating oral film stability. Moreover, the printhead exhibited low ink consumption and minimal material waste. Based on the deposited volume per layer 4.193 ± 0.012 µL, with only 1 mL approximately 59 batches of 4 figures of 1-layer 2 × 4 cm can be printed, underscoring the process's efficiency and scalability. Such low-volume operation is particularly advantageous when working with expensive compounds, such as these hormones, where minimizing waste is critical from both economic and sustainability perspectives [9].

While commercial desmopressin formulations, such as oral tablets (0.1 and 0.2 mg) [30, 31] and sublingual forms (0.120 mg) [13], provide fixed-dose presentations, this novel platform allowed precise dose adjustment and flexibility within a lower range. Recent studies have highlighted orodispersible films (ODFs) [49] and mini-tablets [50] as promising alternatives. In this study, commercially available potato starch substrates were used. Previous studies have shown that printing substrates with a porous architecture, such as edible sheets, are suitable for API-containing inks due to their enhanced liquid uptake. Commercial edible substrates are specifically engineered to provide high ink sorption capacity, and their high porosity has been shown to be a critical factor determining drug loading potential [51]. SEM images corroborated the high porosity of the potato starch substrates, confirming that they were a suitable choice for this type of application. Moreover, the desmopressin-loaded films produced with the novel printhead achieved > 80% drug release within 15 min, matching the performance of developed desmopressin ODFs in previous studies and commercial Minirin® tablets [49] and aligning with prior ODF/mini-tablet studies [50]. Notably, the dissolution testing for the new printed films required significantly lower volumes (20 mL vs. 100–500 mL). Even more striking, disintegration times were reduced to 4–9 s—far below the ≤ 45 s reported for other formulations, enhancing the onset of action. These results support the potential of this approach for oral film development, a promising route for peptide delivery due to buccal vascularization, thin epithelia, and first-pass metabolism avoidance. Additionally, the controlled interaction between the printhead and substrate was confirmed through SEM and TGA, demonstrating film uniformity and structural stability post-deposition. A video in the Supplementary Material provided a detailed view of the contact between the printhead nozzle and the substrate during the printing process.

Furthermore, to achieve the required level of dosing flexibility and personalization, a dedicated software control module was developed to support the integration of the technical pen-based printhead. This custom interface established a robust human–machine interaction (HMI) framework, allowing precise adjustment of key operational parameters, including deposition area, number of layers or printing speed. Although 2 × 4 rectangular pattern was selected in this study since a single layer already provided the clinically relevant dose targeted, the design of alternative geometries is allowed. In this specific scenario, smaller areas would have resulted in doses below the therapeutic range, while substantially larger or significantly different areas would have produced films that were impractical to handle. Nevertheless, in future studies, it will be possible to explore different printed areas and even alternative shapes to expand the range of achievable doses. For example, printing QR or data matrix codes with this technology could be a promising strategy to enhance pharmaceutical traceability or treatment-monitoring [52, 53]. By digitally modulating all these variables, the system ensured reproducible volumetric dosing. The HMI facilitated real-time parameter tuning and process standardization, enabling consistent multi-layered deposition of low-dose formulations.

It should be emphasized that the developed technical pen-based printhead represents a proof-of-concept, and, like all printing technologies, its potential must be further explored across different applications. In this study, the system was tested with two pharmaceutical compounds of distinct natures and drug-free base inks were also explored to evaluate how rheological properties affect deposition. Since the rheological properties of the ink have been thoroughly evaluated and optimized, it can be anticipated that the incorporation of new drugs and formulations with similar properties would also yield satisfactory printing performance and consistent deposition. However, future work could extend these studies to a wider range of drugs and solvent formulations, and explore nozzle diameters other than the 1 mm used, which will impact printability and allow deposition across a broader range of doses and ink viscosities. Moreover, while this work focused on orodispersible films (ODFs) due to their clinical promise and ease of administration, deposition onto non-flat or substrates of different composition remain areas need to be explored to enable deposition onto more complex 3D objects or controlled-release profiles.

From a technical standpoint, the internal design of the printhead requires further refinement to meet pharmaceutical regulatory requirements. As observed in other pharmaceutical printing technologies originated from adapted commercial systems, one of the most critical steps toward clinical implementation is the development of a novel design with dedicated components manufactured from pharmaceutical-grade. This transition is essential to ensure safe contact with drug formulations, minimizing risks of contamination, undesired adsorption phenomena, or material-formulation interactions. In this context, while the present study relies on modified commercial components as a proof-of-concept, future iterations of the printhead would necessarily incorporate pharmaceutical-grade materials to enable safe and regulatory-compliant use. Moreover, manual assembly and cleaning, although facilitated by the modular design, remain labour-intensive and could pose challenges for scalability; adopting disposable, GMP-compliant cartridges similar to those used in clinical SSE studies [54]. In parallel, future iterations of the printhead could incorporate volumetric monitoring approaches to enable real-time assessment of ink consumption during printing and include inline sensors capable of directly monitoring ink flow rate would allow real-time, precise measurement of delivered volume, further strengthening the reliability and control of the deposition process.

Overall, this study presents a proof-of-concept for a novel technical pen-based printhead with a simplified design, demonstrating high accuracy in the deposition of therapeutic microgram-level quantities of a potent hormone. This work is intended as an initial step toward the implementation of technical printheads for precise low-dose pharmaceutical deposition. Future studies will aim to further explore and optimize this printhead, with the ultimate goal of positioning it as a versatile and promising technology within the arsenal for micro-deposition of liquid pharmaceuticals.

## Conclusions

This study successfully integrated a technical pen as a novel printhead in a pharmaceutical 3D printer, enabling the precise and flexible deposition of low doses of desmopressin in oral films. By optimizing printing parameters through a custom software interface, up to four layers of pharma-ink were deposited, achieving therapeutically relevant doses between 33 and 134 µg with high accuracy (99.53–100.21%). The resulting films exhibited rapid drug release, fast disintegration, and demonstrated stability over a one-month period. Additionally, the new printhead operates through gravity, without the need for temperature, pressure or voltage to deposit the ink, thereby preventing molecular stress, which is especially valuable in the case of biopharmaceutical products and other sensitive compounds. These findings support the incorporation of the new printhead into the landscape of advanced manufacturing technologies. Moreover, the increasing demand for accessible, high-precision deposition methods with minimal material waste highlights the advantages of this approach. The technical pen printhead offers a functional inner design, consistent ink deposition, and cost-effective material utilization, making it a promising strategy for the development of pharmaceutical dosage forms and drug-loaded medical devices.

Overall, this work presents a pioneering advancement in pharmaceutical printing by adapting a technical pen onto a pharmaceutical printing platform for controlled drug deposition of a potent hormone. Its combination of precision, accessibility, innovative software integration, and material efficiency positions it as a valuable addition to emerging printing technologies, addressing evolving clinical and manufacturing needs.

## Supplementary Information

Below is the link to the electronic supplementary material.

Supplementary file1 (DOCX 95 KB)

Supplementary file2 (MOV 25192 KB)

## Data Availability

Data generated during and analysed during the current study are available from the corresponding author on reasonable request.
