# Supplementary material for: Technical pen-based printhead for high-precision microdosing of personalized therapies
Source: Drug Deliv Transl Res. 2026 Feb 6;16(10):3746–60. doi: 10.1007/s13346-026-02065-1 (PMC13619802; doi:10.1007/s13346-026-02065-1)
Supplement: Supplementary file 1 [file 13346_2026_2065_MOESM1_ESM.docx]

**Technical Pen-Based Printhead for High-Precision Microdosing of Personalized Therapies**

**Supplementary data**

**Figure S1.** TGA thermal traces for (A) blank film and (B) 4-layers printed film.
